# Conservation Priorities when Species Interact: The Noah's Ark Metaphor Revisited

**DOI:** 10.1371/journal.pone.0106073

**Published:** 2014-09-02

**Authors:** Pierre Courtois, Charles Figuieres, Chloé Mulier

**Affiliations:** INRA-LAMETA, Montpellier, France; University of Kent, United Kingdom

## Abstract

This note incorporates ecological interactions into the Noah's Ark problem. In doing so, we arrive at a general model for ranking *in situ* conservation projects accounting for species interrelations and provide an operational cost-effectiveness method for the selection of best preserving diversity projects under a limited budget constraint.

## Introduction

Weitzman [13] is a milestone in the economic theory of biodiversity. His “Noah's Ark Problem” is not only a modeled metaphor that is helpful to organize thinking on how to face conservation trade-offs with finite resources. It also results in a practical cost-effectiveness methodology that can serve as inspiration to guide conservation policies. The idea is, for each species 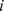, to collect information about: 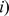 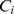, the cost of its protection, 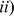 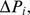 the increase of survival probability resulting from it, 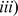 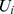, the direct utility of how much we value the species, 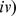 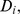 its distinctiveness. From this information, each species is assigned a number 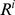 *via* the formula:
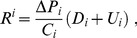
(1)which indicates its rank in conservation priorities. This ranking criterion has a theoretical foundation: it is rooted in a rigorous optimization model ([13], Theorem 4, p. 1295).

This criterion sheds light on real biodiversity issues and has actually been used in several applications. Some of these have led to changes in allocation of conservation funding (e.g., in New Zealand; [9]), and variants have been used to allocate surveillance effort over space (e.g., [8]). Other applications are quoted in [5]. But it is fair to say that this approach is more appropriate for *ex situ* conservation projects - say to build a gene bank or a zoo - rather than to manage a set of interacting species in their natural habitats. This is so because formula (1) uses no information of any kind about the web of life. Yet, in ecosystems, species interact. Some of them compete to share common resources, others develop synergies and mutually enhance each other or they simply pertain to the same trophic chain. Suppose, then, that the conservation authority has information about those ecological interactions, even if it is only under the rudimentary form of survival probability interdependencies. That is, it knows that a marginal increase of survival probability of species 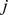 will have an impact 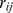 on the survival probability of species 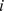. Could this information be used to qualify formula (1) and increase its relevance when it comes to *in situ* conservation trade-offs?

To our knowledge, three recent articles stress the need to account for ecological interactions in Weitzman's diversity concept. They have in common: 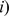 to take into account the ecological interactions *via* interdependent survival probabilities in a simplified version of the Noah's Ark metaphor with two species [1], [11] or three species [12], 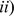 to show that this consideration can reverse the conservation priorities. The key of this note is to provide a general analysis of *in situ* conservation problems considering interdependent survival probabilities. Revisiting Weitzman's optimization problem, we extend his model in order to incorporate species interactions. Our principal output is to forward a general ranking formula that could be used as a rule of thumb for deciding *in situ* conservation priorities under a limited budget constraint.

The sketch of the paper is the following. Section 2 incorporates ecological interactions in Weitzman's parable of Noah's Ark, with any arbitrary number of species. The crux of the section is to provide with a new rule for establishing *in situ* conservation priorities through the expression (12) below that encompasses formula (1) as a special case. The link between this formula and Noah's optimal policy is explained. Section 3 illustrates the relevance of this new formula within a two-species example. We check the robustness of our formula and end the paper with a discussion on the possibility of ranking reversal in relation to three stylized kinds of ecological interactions: *predation*, *mutualism* and *competition*.

## Analysis

The “Noah's Ark Problem” is a parable intended to be a kind of canonical form representing how best to preserve biodiversity under a limited budget constraint. In the initial version of Weitzman's modeled allegory, Noah's decision problem is, for each species 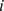, to choose a survival probability between a lower and an upper bound, 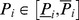, in order to maximize the sum of the *expected diversity function*:
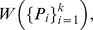
and the *expected utility of the set of species:*



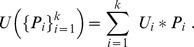



Weitzman devotes much of his paper to defining the expected diversity function 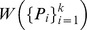 and to explaining its link with the concept of information content (see his Theorem 1, p. 1284). This function could take various specific forms, depending on the way dissimilarity is conceptualized. A precise example, from [13], is discussed in Section 4. In order for our results to remain as general as possible, we simply consider in this paper the class of 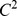 functions, *i.e* whose first and second order derivative both exist and are continuous.

And we assume they admit Hessian matrices that are *nowhere* negative semi-definite, *i.e* there is no admissible 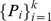 such that the Hessian of 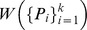 is negative semi-definite at 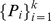. Weitzman's expected diversity function belongs to this class. It encompasses - but is not limited to - functions 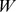 with a positive definite Hessian matrix, *i.e.* that are strictly convex functions.

Now let us take a step away from this initial metaphor, towards reality. Two modifications are brought into the formalism. First, rather than controlling directly the probability of survival 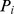 of each species 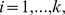 Noah can exert a protection effort within an admissible range, 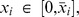 which is interpreted as the controlled increase of survival probability 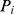 - say that 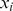 is the increase of survival probability for species 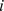 resulting from a protection effort, *e.g.* an investment in a vaccination campaign, the provision of supplementary food, the protection and enhancement of habitat [6]. It is important to distinguish the effort from the change in the survival probability because 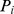 is also determined by other factors, for there are ecological interactions among species. And this is where our second, most important, qualification appears: probabilities of survival are interdependent and the nature of those interactions are known. Nowadays, Noah can rely on the knowledge gained from the new and booming conservation biology literature on *species distribution models* and *population viability analysis*. See for instance[3], [14], [7], or [4] for a recent overview. Note that this literature does not take into account directly of species interactions; it just provides estimates of probabilities in space and time. From there, although applied econometric problems will have to be overcome, correlations between probabilities could be estimated.

A group of experts can measure the marginal impact, say 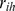, that an increase in the probability of survival of a species 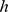 can have on the probability of survival of another species 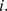 The experts can also appraise the impact of protection efforts on these probabilities. Assume, then, that the relationships between extinction risks are linear. Put differently, a tractable approximation of all those pieces of information can be summarized by the system (2) of linear equations:
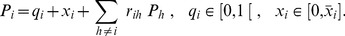
(2)


There are biological and economic factors that determines eligible efforts. Formally, *admissible* ranges of efforts are 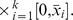 Implicitly, additional efforts beyond the threshold 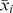 have no effect on the survival probabilities. And we assume:
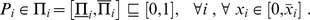



We denote 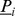 as the survival probability of species 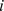 without any conservation efforts, 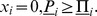 In the absence of natural interactions, which corresponds to the case studied by Weitzman, we have 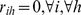. A consequence is that in the very particular case with no ecological interactions and no conservation efforts, species 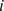 has a probability of survival 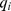. The survival probabilities interval, without ecological interactions, would thus take values ranging from 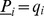 to 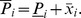


Noah also has to cope with a budget constraint:
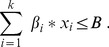
(3)where 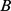 is the total budget to be allocated to conservation - metaphorically, the size of the Ark - and 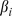 is the cost per unit of effort to preserve species 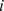.

It is worthwhile making three remarks about this budget constraint. Firstly, it is assumed that changes in extinction probability are a linear function of expenditure. This may be inconsistent in real world applications where the marginal expense needed to reduce extinction risks is increasing. For example, [10] documents that the marginal preservation cost of threatened Australian birds increases when probability of extinction approaches zero. Weitzman rightly defends this linearity assumption as an acceptable approximation when the variation of probability falls in a sufficiently narrow range. But clearly, if costs are non linear and convex functions of efforts, an important qualitative result of our paper could change (Theorem 1 below may not hold any longer). Secondly, as a formal matter one could retrieve Weitzman's model with a simple change of variable, 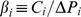 where 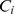 is the cost per unit of increase of survival probability in the range 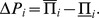 Thirdly, except when ecological interactions are negligible, Noah can increase the probability of survival of any species 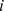 *via* two different channels: a direct one by increasing the protection effort 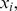 at a cost 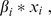 and an indirect one through ecological interactions, due to the protection of another species 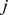, with a cost 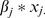


Noah's Ark problem, when ecological interactions are taken into account, is then:
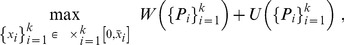
(4)subject to (2) and (3).

It will be convenient subsequently to work with matrix or vector expressions, written in bold characters. For any matrix 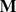, let 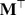 denote its transpose. Further, 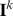 is the 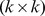 identity matrix, 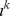 is the 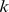 dimensional column vector whose elements are all 1, and we recall the following definition of inequality between two 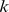-dimensional vectors 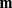 and 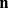 with components 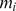 and 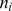 respectively: 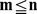 if 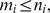 for all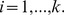 The other basic relationships between vectors are: 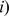 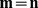 if 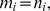
for all 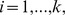 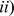 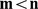 if 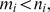 for all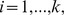 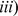 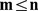 if 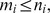 for all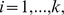 and 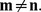 We also need basic matrix operations, “+”, “-” and “*”, that refer to, respectively the addition, the subtraction and the multiplication.

Let us define:
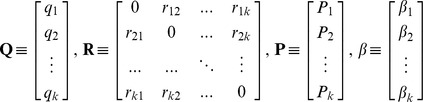


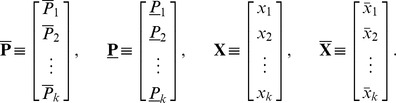



In matrix form, the system (2) reads as:
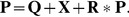
(5)


Throughout this article, we will assume:

**Assumption 1 (INV)** *The matrix* 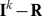 *is invertible*.

Under Assumption (INV), the system (5) can be solved to give:
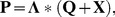
(6)where 
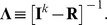


Let 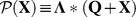 refer to the affine mapping from efforts to probabilities. Survival probabilities without protection policies are therefore:
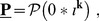
(7)where 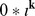 is a vector made of 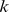 zeroes. Without ecological interactions, 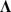 is the identity matrix, 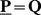 and 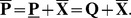


Now we can plug (6) into (4) to get rid of probabilities, and express Noah's problem only in terms of efforts. Define the two *composite* functions, which here are mappings from the values taken by function 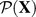 to the set of real numbers:
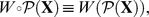


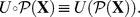



Under Assumption (INV), to each vector 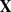 corresponds a unique vector 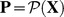. Therefore we can define Noah's problem with ecological interactions, the constrained maximization of a function of protection efforts 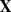:
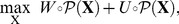
(8)subject to:



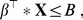
(9)

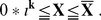
(10)


## Results

Two questions arise: 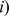 could anything general be said about the solution to the problem expressed by (8), (9), (10)? And 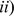, taking a more practical stance, could we engineer a simple rule that approximates the general solution?

### Noah's policy is extreme

Weitzman [13] showed that the solution to Noah's problem lies on the boundary of the efforts set. As the set of constraints is made of linear constraints, the boundary involves corners, e.g. 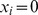 or 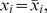 and possibly a segment between two corners, therefore with 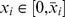 for at most one species. This can be defined as an *extreme policy*. In words, the optimal protection policy gives full protection to a subset of species, partial protection for at most one species, and exposes the remaining species to the risk of no protection.

But what if probabilities are interdependent? We show that when species interact, the optimal solution is also extreme.

**Theorem 1** *The solution to Noah's Ark problem with ecological interactions, defined by (8), (9) and (10), is an extreme policy.*

#### Proof

The proof rests on two pieces of information:

- Noahs' problem is to maximize a continuous function over a compact set, therefore by Weiestrass *extreme value theorem* there exists a solution.
- The Hessian matrix of 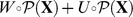 is *not* negative semi-definite, a statement we shall prove below.

Item ii) violates the necessary second order condition for interior solutions to Noah's problem and, in combination with item i), leads to conclude the existence of a solution on the boundary of the efforts set.

In order to prove item ii), because 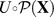 is linear, we just have to ensure that the Hessian matrix of 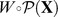 is not negative semi-definite. Recall that 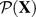 is a 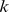-dimensional vector with typical element 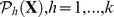, and let 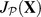 stand for the *Jacobian* matrix:
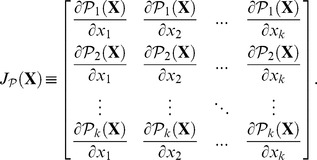



Note that, since each function 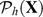 is linear, the Jacobian matrix is made of invariant numbers, so we need not mention the application point 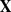 and we can simply refer to the matrix 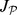.

Denote 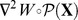 the *Hessian* matrix of 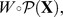 a 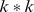 matrix with typical elements 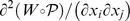. From meticulous derivations of the composite function 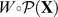, and after simplifications allowed by the linearity of the mapping 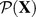, one obtains:
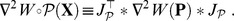



If 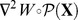 is negative semi-definite, then for any nonzero vector 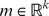 we must have:
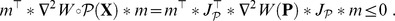



Notice that 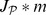 is simply a nonzero 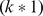 vector, which we may simply call 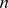. Hence we can rewrite the above inequality as:
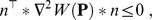
which would mean that 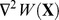 is negative semi-definite, a possibility that has been ruled out by assumption. ▪

### A ranking rule for interacting species

Theorem 1 is a qualitative result, that does not indicate which species should be granted protection and why. This brings us to our second question; it would be welcome to have an explicit and easy-to-use approximation of the general solution. Facing the same problem, this is the practical point of view adopted by [13], which he describes as “the main theme” of his paper (p. 1294). His formula (1) offers a ranking that is not really a solution to the original problem, but rather a first order approximation of an optimal policy. In order to achieve this, he replaces the objective function by its linear approximation. He then obtains a classical linear programming problem, whose solution is to assign grades 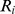 given by formula (1) to species (those grades depend on the model parameters) and order them in decreasing order of importance up to the point where the budget is exhausted. Those grades are exactly the practical ranking Noah is looking for.

We follow the same approach here, *i.e.* we linearize the objective function. The astute reader knows that, in general, such approximations can be seriously misleading [2] and should not be followed blindly. Nevertheless, as proven in Theorem 2 below, there is something special about Noah's problem that makes this practice appropriate here.

Let us denote:
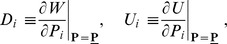
and define the two matrices:



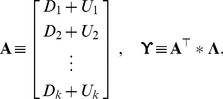



From simple calculations, the linearized problem in matrix form turns out to be:
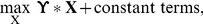
(11)subject to (9) and (10).

As can be observed in the above approximation of Noah's problem, the introduction of ecological interactions changes the “slope” of the objective function to be maximized, which is now 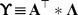 instead of just 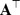. The crux, from the point of view of the present note, is to transform the information about ecological interactions conveyed by matrix 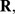 into operational data *via* the matrix 
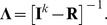
 Given that 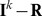 is invertible, the computation of the matrix 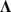 is easily made and if 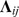 denotes a typical element of 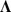, then 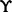 is a 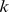-dimensional line vector of the type:
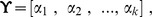
where



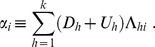



We can now define the “benefit”-cost ratios 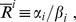 or with explicit reference to relevant information:
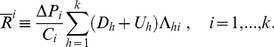
(12)


As it is well-known, the argmax to the linear programming problem (11) is to fully protect the species with the highest grade 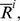 then the species with the second highest grade, and so on and so forth, up to the point where the budget is exhausted. It means that there exists a threshold value 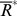 such that all species 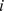 with 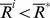 are not embarked in the Ark, whereas those with grade larger than the threshold are all fully protected, except for at most one species with grade exactly equal the cutoff value 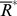 that is only partially protected. Let us call 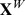 this policy, which can be described formally as follows:
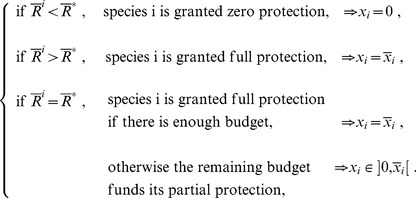
(13)


As shown in Theorem 2 below, 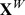 is a first order approximation of the optimal solution to Noah's Ark problem with ecological interactions. Put differently, there is a sense in which expression (12) can be taken for the new practical formula sought to construct *in situ* conservation priorities. Observe that the number assigned to each species 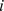 does not depend merely on its own “benefits” but actually on overall “benefits” generated by species 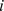 on all the species, 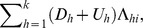 via ecological interactions. Therefore, a species with a strong own interest can be overridden by another, endowed with a less direct interest, but whose importance is enhanced because of its ecological role. Of course, when there are no ecological interactions, 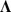 is the identity matrix, with 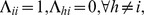 and (12) boils down to Weitzman's original system of grades for species 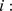


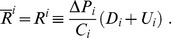



One can ask to what extent can we rely on formula (12) to build a hierarchy among species? Can a conservation policy be based on such an approximation? Baumol and Bushnell in [2] have famously attracted the attention on a number of potential flaws with linear approximations, two of them being important for the problem at hand: *i*) a linear approximation to a nonlinear program need not provide an answer better than a randomly chosen admissible answer, *ii*) only if the objective function behaves monotonically in every variable within the admissible region can we be assured that a linear approximation will yield results which represent an improvement over the point where the linearization is made. Clearly, Noah's objective function does not meet this last condition, for an increase of the effort 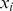 can improve the chances of species 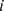 at the expense of another species 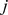 (obviously so when 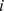 is a predator for 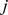).

Still, we can prove the following Theorem which establishes a special interest to the use of a linear approximation in this decision problem:

**Theorem 2** *Consider the Noah's Ark Problem with ecological interactions, defined by (8), (9) and (10), and call 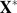 its optimal solution. Then,*

- *the approximation of* 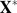 *by* 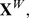 *indicated in (13), offers an improvement compared to the absence of protection*,
- *the approximation error*, 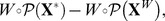 *is no larger than* 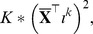 *where* 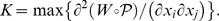

#### Proof. Item i)

The solution proposed in Theorem 2 is inspired from gradient methods used to find optimal solutions based on the property of iterative improvements, like the famous Frank-Wolfe algorithm.

A first step is to replace the objective function by its first order Taylor approximation 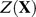 computed at an admissible vector 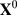 (here at the zero protection vector 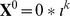). Let us note 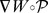 the Gradient, a 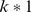 vector with typical elements 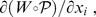 which corresponds actually to the vector 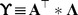 given in the text.

Using those notations:
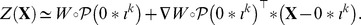



A second step is to find 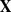 that maximizes 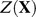 subject to the relevant constraints. Since in 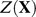 only the term 
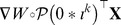
 varies, this step is equivalent to maximize (11) subject to (9) and (10). And the policy 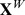 presented in the Theorem 2 is exactly the maximizer of this linear programming problem.

By definition of 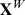, we must have:
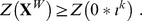


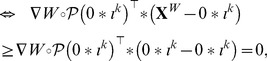
(14)so the vector 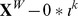 is an ascent direction for 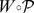. Although this means that the approximation 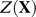 is non decreasing along this direction, it is not guaranteed that the non linear objective will behave similarly, *i.e.* we cannot yet conclude 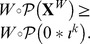


By convexity of function 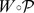 we can write:
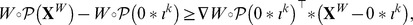
and since we have established in (14):



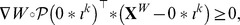
we are led to conclude:



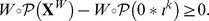



#### Item ii)

Recall that 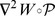 stands for the Hessian matrix of 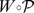. Using Taylor expansions, one can write:



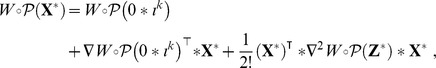
for some admissible vector 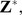 and



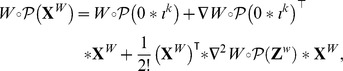
for some admissible vector 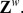 Therefore



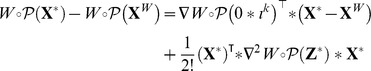





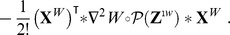



But, by definition of 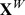


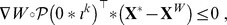
so



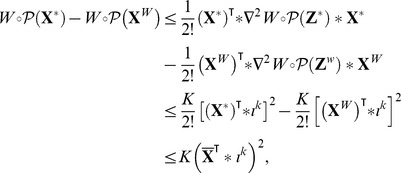
where 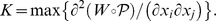 ▪

The upper bound 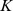 for the approximation error mentioned in the above theorem if of course related to the non-linearity of 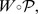 formally captured by the second order derivatives 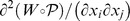. As a matter of interpretation, we can say that the stronger the curvature of 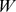 (the stronger preference for diversity if 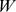 is convex) the larger this upper bound.

### A Two-Species Example: Illustration and Discussion

We close this note with an illustration using a simple two-species example. Let us first study to which extent the consideration of ecological interactions can alter priorities. Assume for simplicity that 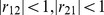. The system (2) becomes:
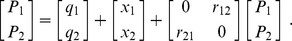



Here the matrix 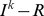 is invertible since 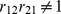.

Solving the system of interactions:
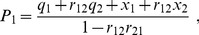
(15)

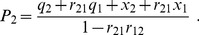
(16)


The grades also can be easily computed. They are:
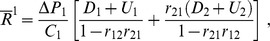


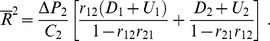



To further simplify, imagine that 
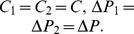
 If ecological interactions are erroneously ignored, formally Noah assigns zero values by mistake to the system of interactions: 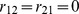. Suppose, without loss of generality, that on this erroneous basis the first species ranks higher:
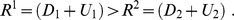



In other words 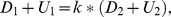
for some 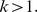


Two questions arise. Could this ranking be reversed once interactions are properly taken into account? And, if the answer is affirmative, why?

When the ranking is reversed:
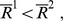


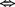


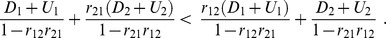



Since 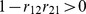, and using 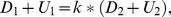 the last inequality is equivalent to:
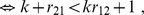


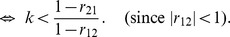



So, a ranking reversal occurs when:
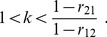
(17)


In order to fix ideas, consider that 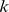 is arbitrarily close to one, *i.e.* the two species provide similar “benefits” and therefore a ranking reversal, if any, is due to the consideration of ecological interactions. Then note that for the above inequality to hold, necessarily 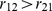, which may occur in various interesting ecological configurations:

- *Predation*: species 1, a predator, feeds on species 2, its prey. So 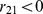 whereas 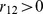. Giving conservation priority to the prey is the most effective way to enjoy the benefits of both species.
- *Mutualism*: for example plant-pollinator interactions, 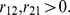 The synergistic relation between those two species is best enhanced by promoting species 2, which has the largest collective marginal impact.
- *Competition*: two species have to share a common resource in the same living area that cannot fully support both populations, hence 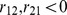, so conservation efforts focus on species 2 because its marginal negative impact is lower.

Let us now examine the robustness of our results by specifying an expected diversity function. Denote 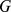 the number of genes jointly owned by the two species whereas 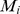 is the total number of genes owned by species 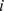. Assume, as in [13] (expression (5)) that the expected (genetic) diversity function takes the following functional form:
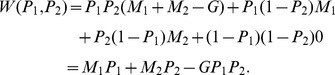



Considering relations (15) and (16) between efforts and probabilities, we obtain:
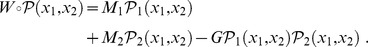



Two questions arise. Can we compare the true solution and the approximate solution? And can we estimate the error due to the approximation of the optimal solution? From Theorem 2, the upper bound on the error due to the approximation can be computed from the Hessian 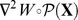. In this two-species example, it is easy to derive the following formulae:
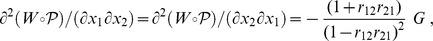


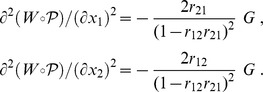



So the upper bound 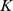 on the approximation error, indicated in Theorem 2, is:
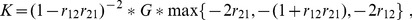
(18)a value which depends only on the number of genes owned jointly by the two species, 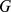, and on the ecological interaction terms, 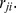


Of course, this is only an upper bound. In some cases, the approximation could also give the exact solution. To illustrate this, assume as before that 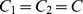, 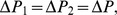 that utilities are identical, 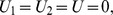 and the upper bounds on efforts are the same for the two species, 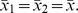 Assume also that the total budget can cover the protection cost of only one species, 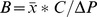. Noah then has to choose among two extreme policies, the first one 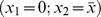 that provides the following expected diversity:
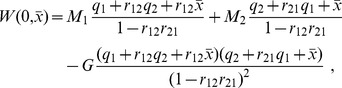
and the second one 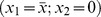 with expected diversity:



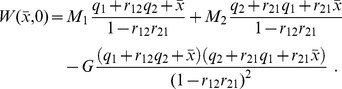



It is optimal to protect species 2 if:
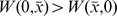


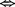


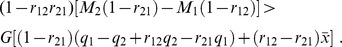



In the particular case where 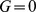, then 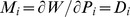, and the above condition boils down to a very simple expression:
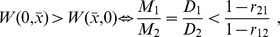
a condition which is also necessary for the approximated solution to select species 2 (remember condition (17)). It comes as no surprise that the optimal solution and its approximation concide, since when 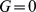 the upper bound on the approximation error is zero, as can be seen from expression (18).
